# E-Cigarette Use and Incident Hypertension: Considerations for Interpretation

**DOI:** 10.1016/j.jacadv.2026.102802

**Published:** 2026-05-12

**Authors:** Davide Campagna, Giulio Geraci, Yusuff Adebayo Adebisi

**Affiliations:** a Center of Excellence for Acceleration of HArm Reduction (CoEHAR), University of Catania, Catania, Italy; b Department of Clinical & Experimental Medicine, University of Catania, Catania, Italy; c UOC MCAU, University Teaching Hospital "Policlinico-S.Marco", University of Catania, Catania, Italy; d Faculty of Medicine and Surgery, "Kore" University of Enna, Enna, Italy; e Nuffield Department of Population Health, University of Oxford, Oxford, United Kingdom; f College of Social Sciences, University of Glasgow, Glasgow, Scotland, United Kingdom

We read the analysis by Lee et al from the Hispanic Community Health Study/Study of Latinos reporting an association between baseline “ever” e-cigarette use and incident hypertension over 6 years of follow-up, despite no association with prevalent hypertension and no detectable differences in longitudinal blood pressure (BP) change.^1^ The question is important, but several aspects of exposure measurement, confounding, and result patterns warrant cautious interpretation.

First, the primary exposure, “ever e-cigarette use,” defined as having used an e-cigarette “even once,” combines heterogeneous behaviors into a single category. Even when subdivided, “former” users (ever use but no past-30-day use at baseline) showed elevated incident hypertension risk (HR: 1.43; 95% CI: 1.07-1.93). Because recency, duration, and intensity are not measured and use is assessed only at baseline, temporality and dose-response cannot be evaluated, and proposed mechanisms involving repeated BP spikes cannot be directly tested.

Second, ever e-cigarette users had markedly higher combustible smoking rates than never e-cigarette users (52.1% vs 12.7% current smokers). Adjustment using categorical smoking status (never/former/current) may not fully capture smoking intensity, cumulative exposure (eg, pack-years), or changes during follow-up, all of which are relevant to hypertension risk. When the combination of ever e-cigarette and combustible cigarette use was examined, dual ever users showed significant associations with incident hypertension, whereas exclusive e-cigarette users did not. This pattern is consistent with the possibility that combustible cigarette exposure contributes substantially to the observed associations.^2^

Third, the findings are sensitive to outcome definition and modeling choices. Ever e-cigarette use was not associated with prevalent hypertension at baseline (adjusted prevalence ratio 0.86; 95% CI: 0.73-1.02), yet was associated with incident hypertension during follow-up (adjusted HR: 1.49; 95% CI: 1.14-1.95). Unadjusted Kaplan-Meier curves showed no significant differences by e-cigarette status (log-rank *P* > 0.05), and longitudinal changes in measured BP from visit 2 to visit 3 did not differ significantly across groups. Notably, the association was observed in multivariable Cox models and attenuated using the contemporary hypertension threshold (≥130/80 mm Hg), with loss of statistical significance for ever vs never users (HR: 1.20; 95% CI: 0.89-1.61). These patterns underline the need for caution and raise the possibility of residual confounding and/or exposure misclassification.

Fourth, stronger associations in younger (<50 years: HR: 1.78) vs older participants (≥50 years: HR: 0.82; p-interaction = 0.024) appears inconsistent with expected biological patterns. Nicotine-induced vasoconstriction and endothelial dysfunction would plausibly affect older adults (with reduced vascular reserve) at least as strongly, if not more, suggesting possible residual confounding or selection bias by age group.

Fifth, among the 6,306 participants without baseline hypertension, only 89 were current e-cigarette users; just 18 developed hypertension during follow-up. The reported HR of 1.89 (95% CI: 1.12-3.18) rests on fewer than 20 events, raising stability concerns. Multiple subgroup analyses without adjustment for multiple comparisons increase false-positive risk.

Finally, the discussion should better situate results within clinical substitution evidence. A living systematic review reported that nearly two-thirds of analyses showed no significant BP differences with e-cigarette substitution and noted clinically relevant systolic BP reductions after 1 year in studies of participants with hypertension.^3^ One prospective study in hypertensive smokers switching to e-cigarettes reported improved BP control.^4^ A randomized trial in chronic smokers switching from tobacco cigarettes to e-cigarettes reported improved vascular function within 1 month.^5^

We suggest these findings be interpreted cautiously as hypothesis-generating until replicated with repeated, granular, time-updated measures of e-cigarette use and detailed smoking history that better distinguish exclusive e-cigarette use from dual use.
